# Predicting overall survival in glioblastoma patients using machine learning: an analysis of treatment efficacy and patient prognosis

**DOI:** 10.3389/fonc.2025.1539845

**Published:** 2025-04-09

**Authors:** Razvan Onciul, Felix-Mircea Brehar, Adrian Vasile Dumitru, Carla Crivoi, Razvan-Adrian Covache-Busuioc, Matei Serban, Petrinel Mugurel Radoi, Corneliu Toader

**Affiliations:** ^1^ Department of Neurosurgery, “Carol Davila” University of Medicine and Pharmacy, Bucharest, Romania; ^2^ Neurosurgery Department, Emergency University Hospital, Bucharest, Romania; ^3^ Department of Neurosurgery, Clinical Emergency Hospital “Bagdasar-Arseni”, Bucharest, Romania; ^4^ Department of Pathology, University Emergency Hospital Bucharest, Carol Davila University of Medicine and Pharmacy, Bucharest, Romania; ^5^ Department of Computer Science, Faculty of Mathematics and Computer Science, University of Bucharest, Bucharest, Romania; ^6^ Department of Vascular Neurosurgery, National Institute of Neurology and Neurovascular Diseases, Bucharest, Romania

**Keywords:** machine learning, prognostic biomarkers, explainable AI, survival prediction, clinical decision support, personalized medicine, predictive modeling

## Abstract

**Introduction:**

Glioblastoma (GBM), the most aggressive primary brain tumor, poses a significant challenge in predicting patient survival due to its heterogeneity and resistance to treatment. Accurate survival prediction is essential for optimizing treatment strategies and improving clinical outcomes.

**Methods:**

This study utilized metadata from 135 GBM patients, including demographic, clinical, and molecular variables such as age, Karnofsky Performance Status (KPS), MGMT promoter methylation, and EGFR amplification. Six machine learning models—XGBoost, Random Forests, Support Vector Machines, Artificial Neural Networks, Extra Trees Regressor, and K- Nearest Neighbors—were employed to classify patients into predefined survival categories. Data preprocessing included label encoding for categorical variables and MinMax scaling for numerical features. Model performance was assessed using ROC-AUC and accuracy metrics, with hyperparameters optimized through grid search.

**Results:**

XGBoost demonstrated the highest predictive accuracy, achieving a mean ROC-AUC of 0.90 and an accuracy of 0.78. Ensemble models outperformed simpler classifiers, emphasizing the predictive value of metadata. The models identified key prognostic markers, including MGMT promoter methylation and KPS, as significant contributors to survival prediction.

**Conclusions:**

The application of machine learning to GBM metadata offers a robust approach to predicting patient survival. The study highlights the potential of ML models to enhance clinical decision-making and contribute to personalized treatment strategies, with a focus on accuracy, reliability, and interpretability.

## 1 Introduction

Glioblastoma (GBM) remains the most aggressive and fatal primary brain tumor in adults, with a median survival of just 15 months despite advancements in surgical, radiotherapeutic, and chemotherapeutic interventions. This stark prognosis is driven by GBM’s inherent heterogeneity and resistance to treatment, making precise prognostic assessments critical yet elusive (1). Traditional methods often fail to fully capture the intricate biological and clinical interplay that shapes patient outcomes, paving the way for innovative computational approaches to address this gap (2).

Machine learning (ML) has introduced a transformative perspective in predicting GBM survival by leveraging multidimensional data. Recent advancements in ML enable the integration of diverse inputs, such as genetic markers, epigenetic profiles, and clinical variables, into predictive models (3). These systems have moved beyond conventional tools, offering individualized survival estimates that reflect the complexity of GBM biology. Multimodal approaches have proven especially impactful, synthesizing molecular data with imaging and clinical parameters to deliver nuanced, patient-specific insights (4).

Radiomics, a rapidly evolving field, has further enhanced survival prediction by unlocking the potential of standard imaging techniques. Through the extraction of high-dimensional features from MRI scans, radiomics reveals patterns linked to tumor progression and microenvironment characteristics (5). When combined with deep learning, these features have become powerful prognostic indicators, offering an unprecedented level of precision and interpretability. Such approaches are not only predictive but also uncover new biological connections, linking imaging characteristics to molecular and clinical outcomes (6).

In parallel, machine learning models have demonstrated the ability to address challenges related to data variability and interpretability. By leveraging advanced algorithms, these models can effectively identify key survival factors and achieve robust predictive accuracy. This approach has facilitated the identification of critical variables, offering actionable insights to support the development of tailored treatment strategies (7).

Despite these advances, challenges remain, including data imbalance and the need for broader validation across diverse cohorts. Innovative solutions, such as data augmentation and transfer learning, are actively addressing these barriers, pushing the boundaries of what ML can achieve in clinical settings (8).

This study builds on these novel developments, harnessing advanced ML methods to create predictive models that incorporate diverse data modalities. By addressing existing limitations and enhancing interpretability, this work aims to improve survival predictions and contribute to a more personalized approach to GBM management.

## 2 Background

GBM is a highly aggressive brain tumor with poor outcomes, presenting unique challenges for accurate prognosis and treatment planning. Traditional approaches often struggle to account for the complex biological and clinical variability inherent to GBM, necessitating advanced methodologies to improve survival predictions (9). ML has emerged as a promising tool to address these gaps, leveraging diverse datasets to provide more personalized and precise prognostic insights.

### 2.1 Advances in machine learning for GBM prognosis

The use of radiomics, a field focused on extracting detailed imaging features, has significantly enhanced the prognostic capabilities of ML. By analyzing high-dimensional data from MRI scans, radiomics enables models to detect subtle patterns related to tumor behavior and patient outcomes (10). For example, ML models incorporating imaging-derived metrics, such as texture and shape features, have demonstrated considerable accuracy in predicting survival times. These approaches are especially valuable for their non-invasive nature and ability to complement existing clinical evaluations (11).

Beyond imaging, molecular profiling has emerged as a critical component in GBM prognosis. Genomic and transcriptomic data have proven essential for identifying key survival markers, such as MGMT promoter methylation and IDH mutation status. The integration of molecular data into ML frameworks has facilitated more nuanced stratifications of patient outcomes, offering insights that align closely with tumor heterogeneity (12). Multi-omics approaches, which combine molecular, proteomic, and clinical information, further enhance predictive accuracy by capturing a holistic view of the disease (13).

Multimodal frameworks that combine radiomic, molecular, and clinical data have demonstrated exceptional potential for survival prediction. Ensemble learning algorithms, such as gradient boosting and random forests, excel in synthesizing disparate data types to uncover predictive patterns. These models are particularly effective in handling data variability and prioritizing key survival factors, making them reliable tools for GBM prognosis (14).

### 2.2 Addressing challenges in ML applications

Despite the advancements, several obstacles remain in applying ML to GBM prognosis. One of the most pressing issues is class imbalance, where long-term survival categories are underrepresented in datasets (15). Techniques such as Synthetic Minority Oversampling (SMOTE) have been employed to address this imbalance, enhancing model robustness and improving predictions for minority classes. Additionally, the interpretability of complex ML models poses a challenge for clinical adoption (16). Emerging tools like SHapley Additive exPlanations (SHAP) and Local Interpretable Model-agnostic Explanations (LIME) are bridging this gap by elucidating the contributions of individual features to model predictions, fostering greater trust and usability in clinical contexts (17, 18).

### 2.3 Relevance of current study

This study aims to build on these advancements by integrating molecular, and clinical data into an advanced ML framework to improve GBM survival predictions. By addressing challenges such as data imbalance and interpretability, this research seeks to develop robust, transparent models that are both accurate and clinically applicable. The results aim to contribute to more personalized approaches in GBM management, advancing the integration of ML into routine oncology practice.

## 3 Data description

### 3.1 Data Collection and Filtering

The dataset for this study began with 17 columns, encompassing various clinical, demographic, molecular, and treatment-related variables. To refine the dataset, we focused on patients who underwent surgical resection and had either radiotherapy or chemotherapy as part of their treatment. After applying these inclusion criteria, the final cohort consisted of 135 patients. This carefully filtered dataset provided a robust foundation for analyzing survival outcomes and training machine learning models.

### 3.2 Dataset overview

The dataset includes features essential for understanding glioblastoma prognosis, grouped into the following categories:

1. Demographic Features:

Age: The patient’s age at diagnosis, recorded as a continuous variable.
Gender: A categorical variable (male/female), converted into numerical format for analysis.

2. Clinical Features:

Karnofsky Performance Status (KPS): A score that evaluates the patient’s physical ability and functional independence.
Overall Survival (OS): The primary outcome variable, categorized into five survival classes:

• 0–2 months

• 3–8 months

• 9–18 months

• 19–24 months

• More than 24 months

3. Treatment Details:

Radiotherapy: Indicates whether the patient received radiotherapy. Chemotherapy: Indicates whether the patient underwent chemotherapy.
Surgical Resection: Indicates whether the patient had a surgical procedure to remove the tumor.

4. Molecular Biomarkers:

MGMT Promoter Methylation: A binary marker associated with the tumor’s sensitivity to treatment.
EGFR Amplification: A binary marker linked to tumor growth and progression.

Features for Machine Learning Models

From the dataset, a selection of key features was made to train machine learning models effectively:

- Demographic Data: Age and gender.
- Clinical Features: KPS score and categorized overall survival as the target variable.
- Treatment Information: Whether the patient received radiotherapy, chemotherapy, or surgical resection.
- Molecular Markers: MGMT promoter methylation and EGFR amplification.

This selection captures a holistic view of each patient, ensuring that the models are equipped to analyze the multifaceted factors influencing glioblastoma outcomes.

### 3.3 Data preparation

To prepare the data for machine learning, several preprocessing steps were implemented:

- Categorical Encoding: Variables like gender and molecular biomarkers were converted into numerical values for compatibility with ML algorithms.
- Normalization: Continuous variables such as age, KPS score, and imaging features were normalized to ensure all inputs had comparable scales.
- Classification of Survival: The overall survival variable was divided into discrete categories, enabling classification-based machine learning methods.

### 3.4 Final dataset characteristics

The final dataset comprised 135 patients with selected features spanning demographic, clinical, molecular, and imaging data. This dataset provided the basis for developing machine learning models aimed at accurately predicting survival outcomes and aiding in personalized treatment strategies for glioblastoma patients.

## 4 Data preprocessing

In the dataset derived from the clinical study on glioblastoma, an essential preprocessing step involved converting all textual or categorical variables into numeric formats. This transformation was accomplished using label encoding, a technique where each unique text value in a column is assigned a numerical label. This process included encoding variables that describe medical interventions, like types of surgery or chemotherapy, as well as genetic features such as MGMT promoter methylation status and EGFR amplification.

Additionally, the numerical variables in the dataset, specifically age and Karnofsky Performance Status (KPS), underwent scaling using the MinMax method. This method transforms the data into a specified range, for us 0 to 1, by subtracting the minimum value of each feature and then dividing by the range of the feature. The formula for MinMax scaling is:


$$Scaled\;Value=\frac{Original\;Value-Minimum\;Value}{Maximum\;Value-Minimum\;Value}$$

Scaling is an important method in data preprocessing because it brings uniformity to different features, ensuring that no feature dominates others in magnitude, which can affect the performance of many machine learning models. Second, it improves the convergence speed of the algorithm because most machine learning algorithms perform better when the numerical input values vary similarly.

By normalizing the data across the entire dataset through these methods, the processed data becomes more suitable for predictive modeling, enhancing the efficiency and accuracy of the machine learning models developed from this dataset. These steps ensure that the data analysis is robust, providing reliable insights into the treatment outcomes and potential prognostic factors in glioblastoma patients.

### 4.1 Data preparation and survival analysis framework

In the clinical dataset focusing on glioblastoma, the primary outcome of interest is OS, which quantifies the duration a patient lives following their diagnosis. This target variable is pivotal for assessing the efficacy of various treatments and for making predictions about patient prognosis.

The dataset categorizes Overall Survival into five distinct classes based on the number of months a patient survives post-diagnosis, starting with the 0-2 months range, followed by 3-8 months, then 9-18 months, 19-24 months, and finally, more than 24 months.

These classes enable the machine learning models to handle survival data as a categorical variable, which simplifies the modeling of survival distributions across different patient groups.

The training set of the dataset reveals a distribution of patients across the survival classes, with 7 patients in Class 0 (0-2 months), 37 patients in Class 1 (3-8 months), 46 patients in Class 2 (9-18 months), and 2 patients each in Class 3 (19-24 months) and Class 4 (more than 24 months).

This distribution helps in understanding the model’s learning capacity across a varied range of survival outcomes, although it highlights an imbalance in the dataset with fewer representatives in the longer survival categories.

The test set, used to evaluate the performance of the predictive model, showing 5 patients in Class 0 (0-2 months), 16 patients in Class 1 (3-8 months), 16 patients in Class 2 (9-18 months), 2 patients each in Class 3 (19-24 months) and Class 4 (more than 24 months).

This distribution indicates how the model will be tested against unseen data, offering insights into its generalized performance across different survival times.

### 4.2 Models training

In the study focusing on glioblastoma patient survival, six types of machine learning models were employed to predict outcomes. These models include Artificial Neural Networks (ANN), Extra Trees Regressor (ETR), K-Nearest Neighbors (KNN), Random Forest (RF), Support Vector Machines (SVM), and XGBoost Regressor (XGBR). Each model brings a distinct approach to handling the data and making predictions, leveraging their unique strengths to potentially improve the accuracy of survival time predictions.

All models underwent a fine-tuning process to optimize their parameters, ensuring the best possible performance (**Table 1**). This fine-tuning was performed using grid search, a systematic approach to hyperparameter optimization. Grid search iteratively evaluates combinations of hyperparameter values to identify the configuration that delivers the best performance for each model. The process ensures that the models are well-calibrated to the dataset, avoiding underfitting or overfitting.

**Table 1:** Presents the optimized hyperparameter settings for each of the six machine learning models used in this study.

Classifier	Hyperparameters
ANN	Hidden Layers: - First layer: 32 neurons, ReLU activation - Second layer: 32 neurons, ReLU activation Output Layer: 5 neurons, Softmax activation Optimizer: Adam Loss Function: Categorical Crossentropy Epochs: 50 Batch Size: 16
SVM	Kernel: ‘poly’
XGB	Objective: binary:logistic Column Sample By Tree: 0.5 Learning Rate: 0.1 Max Depth: 100 Alpha: 1 Number of Estimators: 50
RF	n_estimators: 8
ETR	n_estimators: 5
KNN	n_neighbors: 30

The ANN model, for example, consists of two hidden layers with ReLU activation functions and was trained using the Adam optimizer and categorical crossentropy loss. The XGBoost model was fine-tuned with parameters such as a maximum tree depth of 100 and a learning rate of 0.1, while simpler models like KNN and Random Forest used optimized settings for the number of neighbors and estimators, respectively. These fine-tuned configurations ensure that each model performs optimally when predicting glioblastoma patient survival outcomes.

## 5 Results

We evaluated the performance of six machine learning classifiers by analyzing their predictive accuracy and ROC-AUC on the test set. All reported performance metrics, including ROC-AUC and accuracy, were derived from the test set, ensuring the evaluation reflects the models’ ability to generalize to unseen data. The models were trained on the training set, and hyperparameters were optimized using grid search to avoid overfitting (**Table 2**).

**Table 2:** Provides a boxplot comparison of the Receiver Operating Characteristic Area Under the Curve (ROC-AUC) performance for all classifiers used in the study.

Classifier	ROC - AUC	Accuracy
ANN	Mean: 0.73 Standard deviation: 0.15	0.68
SVM	Mean: 0.84 Standard deviation: 0.06	0.63
XGB	Mean: 0.90 Standard deviation: 0.07	0.78
RF	Mean: 0.80 Standard deviation: 0.12	0.66
ET	Mean: 0.82 Standard deviation: 0.19	0.78
KNN	Mean: 0.79 Standard deviation: 0.14	0.54

ROC-AUC is a key metric for evaluating the predictive accuracy of machine learning models, particularly for classification tasks involving imbalanced datasets.

**Figure 1:**
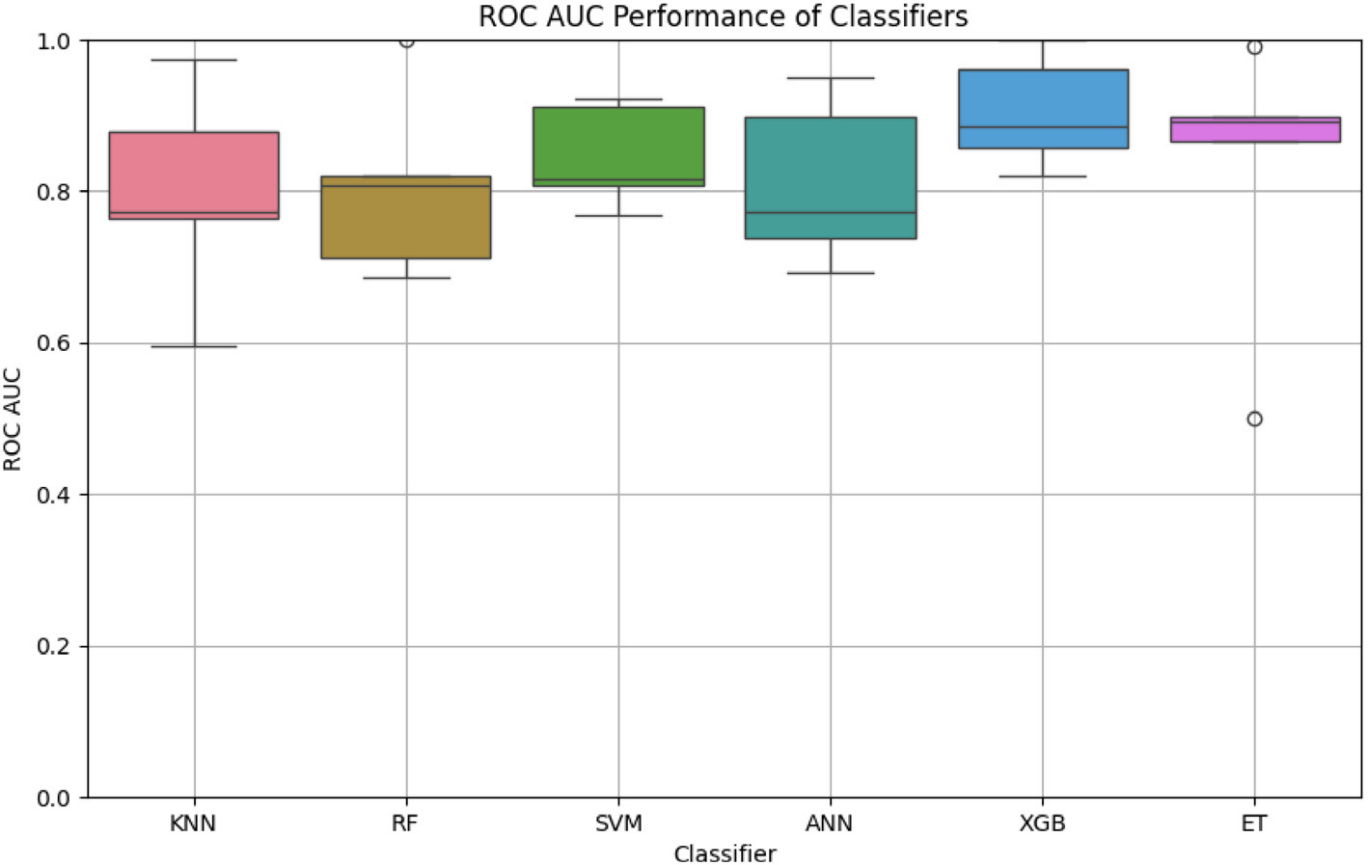
The boxplot highlights the variation in ROC-AUC scores for each classifier. XGB demonstrates the highest median ROC-AUC score with minimal variability, followed by ET and SVM. In contrast, KNN exhibits higher variability and lower performance compared to other classifiers, suggesting sensitivity to the dataset’s features.

In this study, we evaluated the robustness and efficiency of six machine learning classifiers by analyzing their ROC AUC scores across multiple iterations, possibly obtained through cross-validation or bootstrap resampling (**Figure 1**). This approach helps to gauge the performance stability and effectiveness of each classifier in predictive tasks.

**Figure 2:**
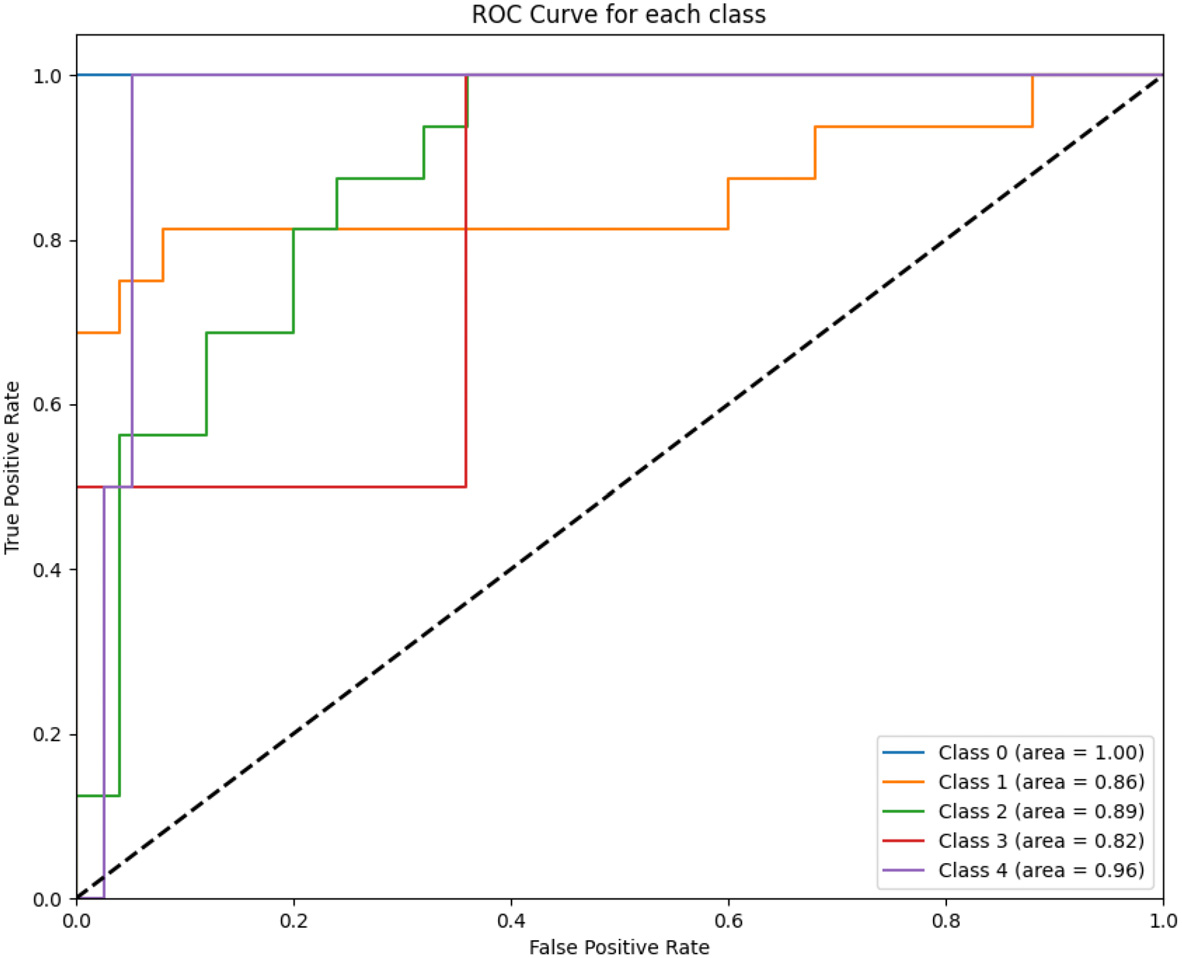
The figure highlights the model’s performance in distinguishing between survival classes. The area under the curve (AUC) values for each class are annotated in the legend. Class 0 achieves perfect discrimination with an AUC of 1.00, while intermediate survival classes show moderate performance (e.g., Class 2 with an AUC of 0.89).

**Figure 2** displays the ROC curves for XGBoost for predicting survival across all five classes (0–2 months, 3–8 months, 9–18 months, 19–24 months, and more than 24 months) using one of the evaluated models. The ROC curve illustrates the trade-off between the true positive rate (sensitivity) and the false positive rate for each survival class.

The best results were achieved using an XGBoost algorithm, which attained an average ROC-AUC of 0.90 with a standard deviation of 0.07 and an accuracy of 0.78 on the test data. The next best outcomes were observed with an ET classifier, which demonstrated an ROC-AUC mean of 0.82, a standard deviation of 0.19, and achieved an accuracy of 0.78. Following the XGBoost and Ensemble Tree classifiers, the Support Vector Machine (SVM) algorithm also showed promising results with a mean ROC-AUC of 0.84 and a low standard deviation of 0.06, although its accuracy on the test data was slightly lower at 0.63. The RF classifier, with a mean ROC-AUC of 0.80 and a standard deviation of 0.12, achieved an accuracy of 0.66, demonstrating robustness albeit with a bit more variability in its performance compared to SVM.

**Figure 3:**
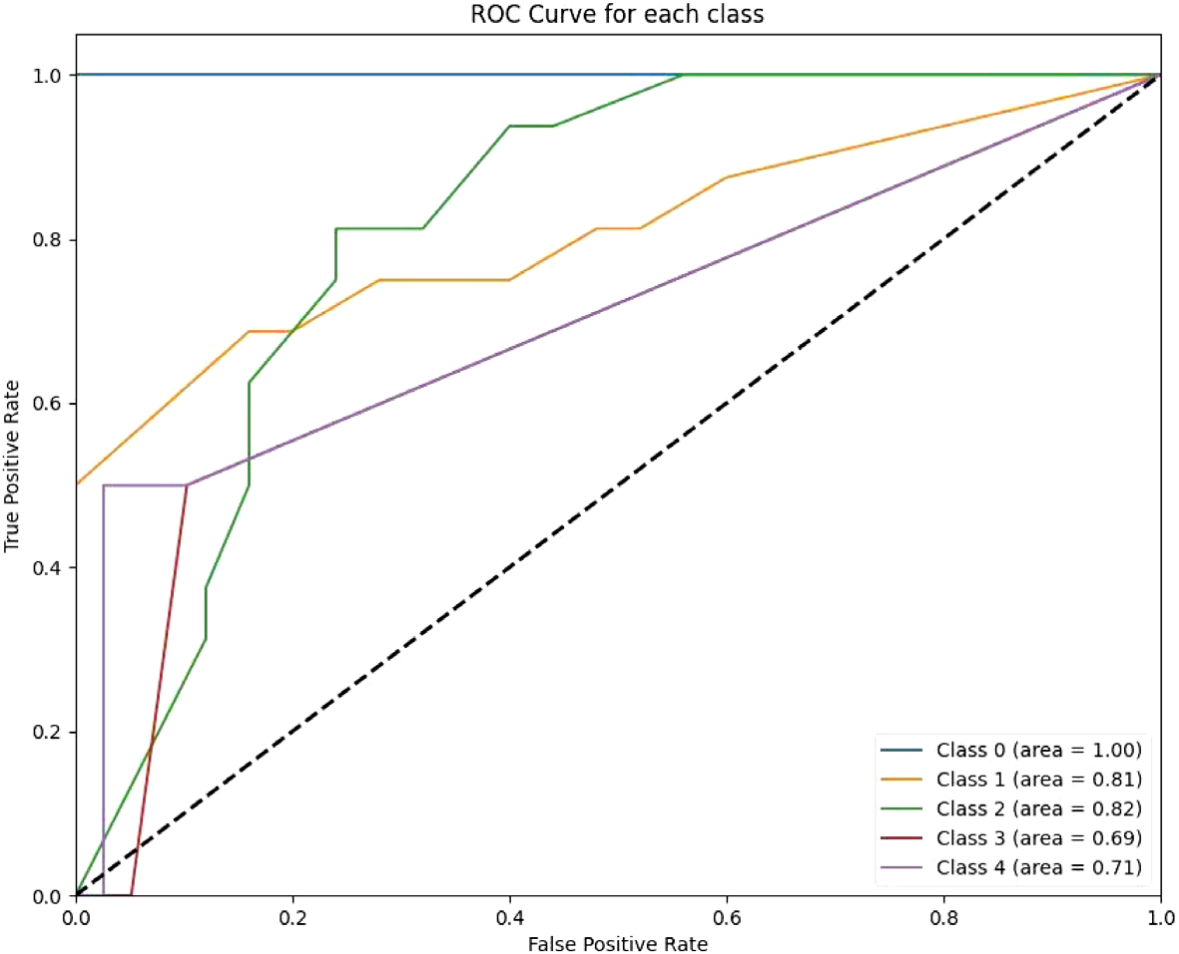
The RF ROC curves display class-wise prediction accuracy, with Class 0 achieving the highest AUC of 1.00. Intermediate classes (e.g., Class 1 and Class 2) demonstrate moderate predictive performance, with AUC values ranging between 0.81 and 0.82. The lower AUC for Class 3 (0.69) and Class 4 (0.71) indicates difficulty in distinguishing between these classes.

**Figure 4:**
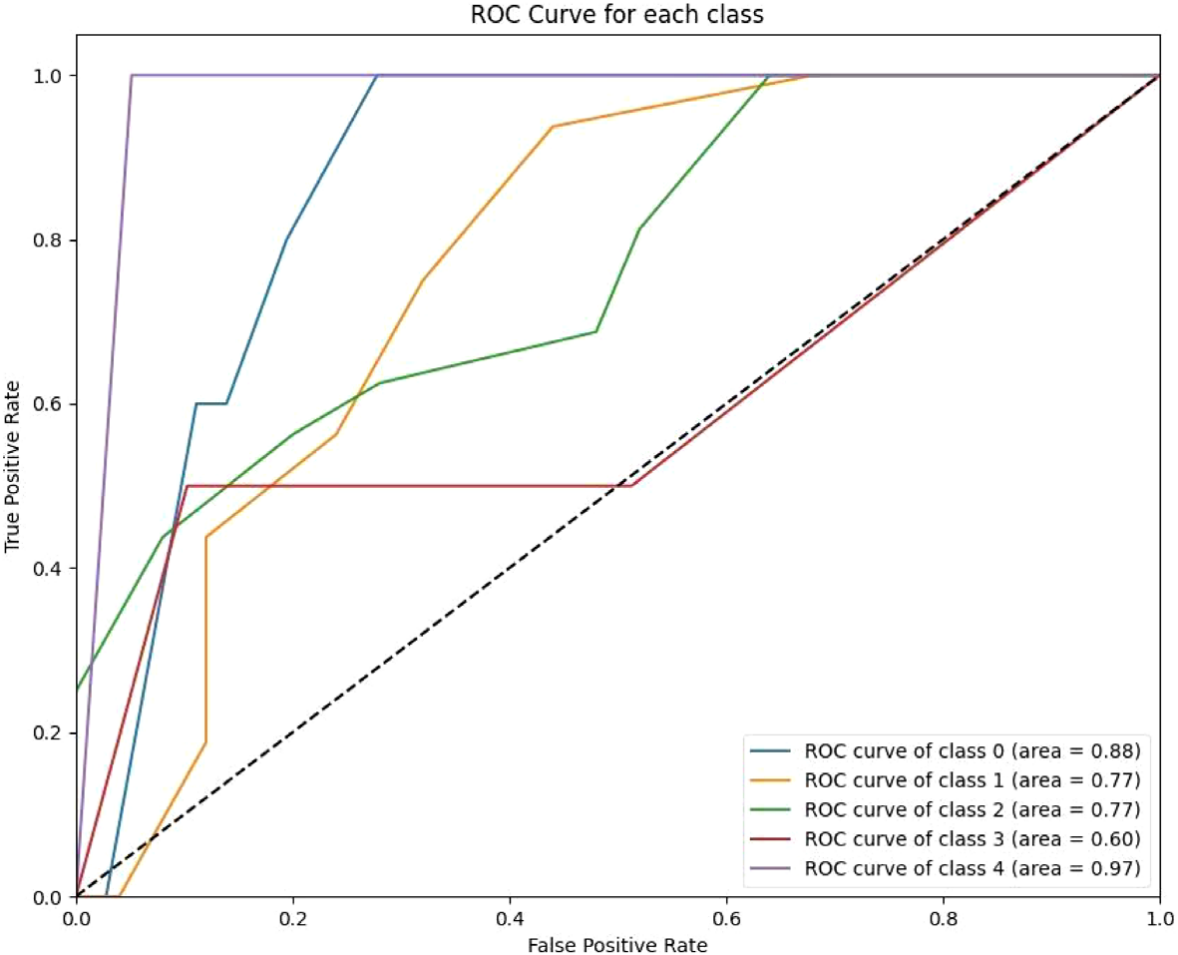
The KNN ROC curves indicate variability in the model’s performance across classes. Class 0 achieves a high AUC of 0.87, reflecting strong predictive capability for short-term survival. However, other classes, such as Class 3 (AUC 0.79) and Class 4 (AUC 0.94), show modest improvements over previous models.

**Figure 5:**
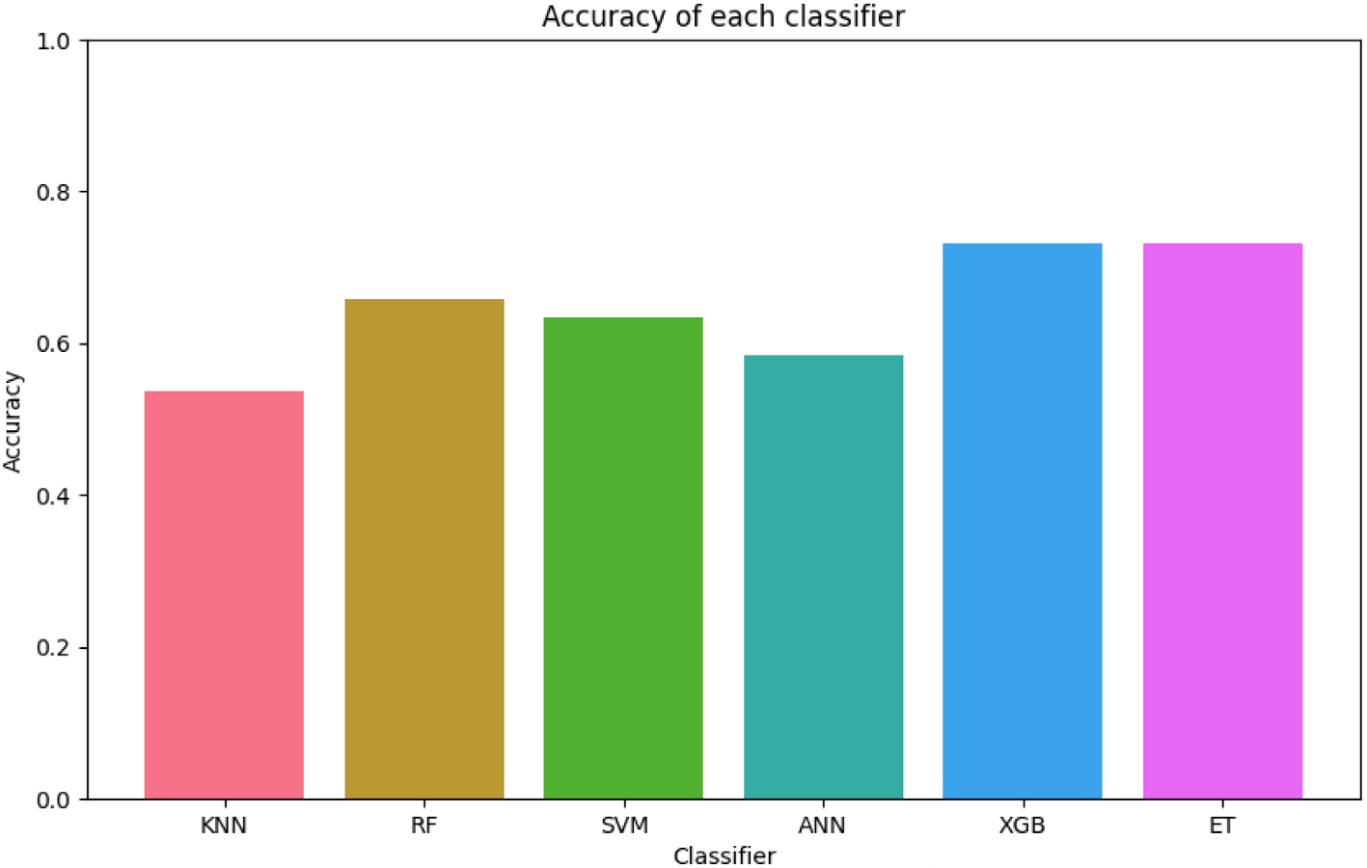
The figure shows that XGB and ET achieve the highest accuracy, with values close to 0.8, indicating robust predictive capabilities. In contrast, KNN records the lowest accuracy at approximately 0.5, highlighting its limitations for this dataset. The figure underscores the overall reliability of tree-based ensemble models compared to simpler classifiers such as KNN.

The ANN model recorded a mean ROC-AUC of 0.73 and the highest standard deviation of 0.15 among the classifiers, alongside an accuracy of 0.68 on the test data, indicating less consistency in its predictive ability. Lastly, the KNN algorithm, while it had a decent mean ROC-AUC of 0.79 and a standard deviation of 0.14, showed the lowest test accuracy of 0.54, suggesting it might not be as effective in this particular setting compared to the other models (**Figures 3**–**5**).

These findings highlight the efficacy of XGB and ET classifiers in handling complex predictive tasks, with XGB slightly outperforming others in terms of stability and overall performance.

**Figure 6:**
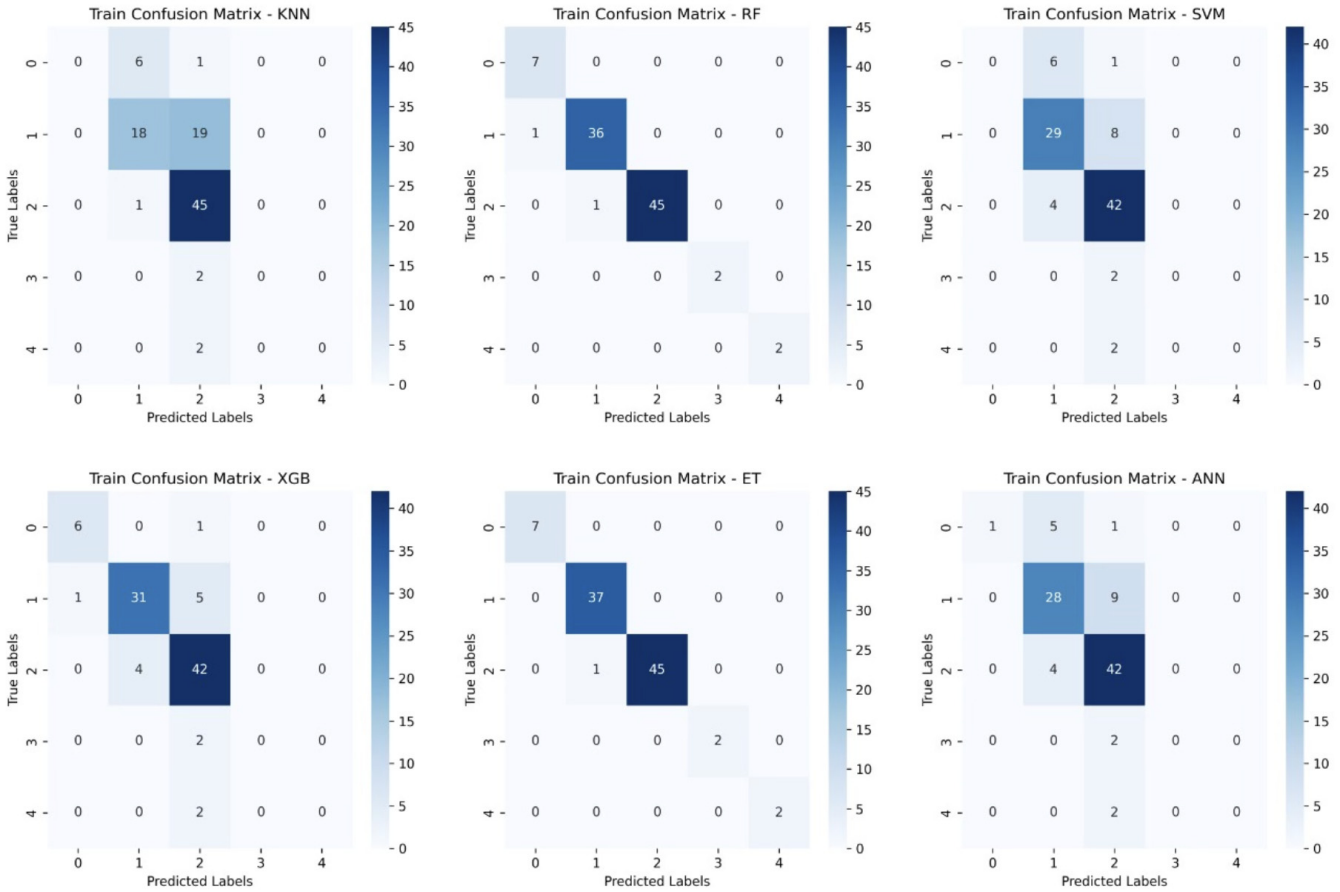
Training Confusion Matrices for Six Machine Learning Models. This figure presents the confusion matrices for six machine learning models (KNN, Random Forest (RF), Support Vector Machine (SVM), XGBoost, Extra Trees (ET), and Artificial Neural Networks (ANN)) trained on glioblastoma patient survival classification.

**Figure 7:**
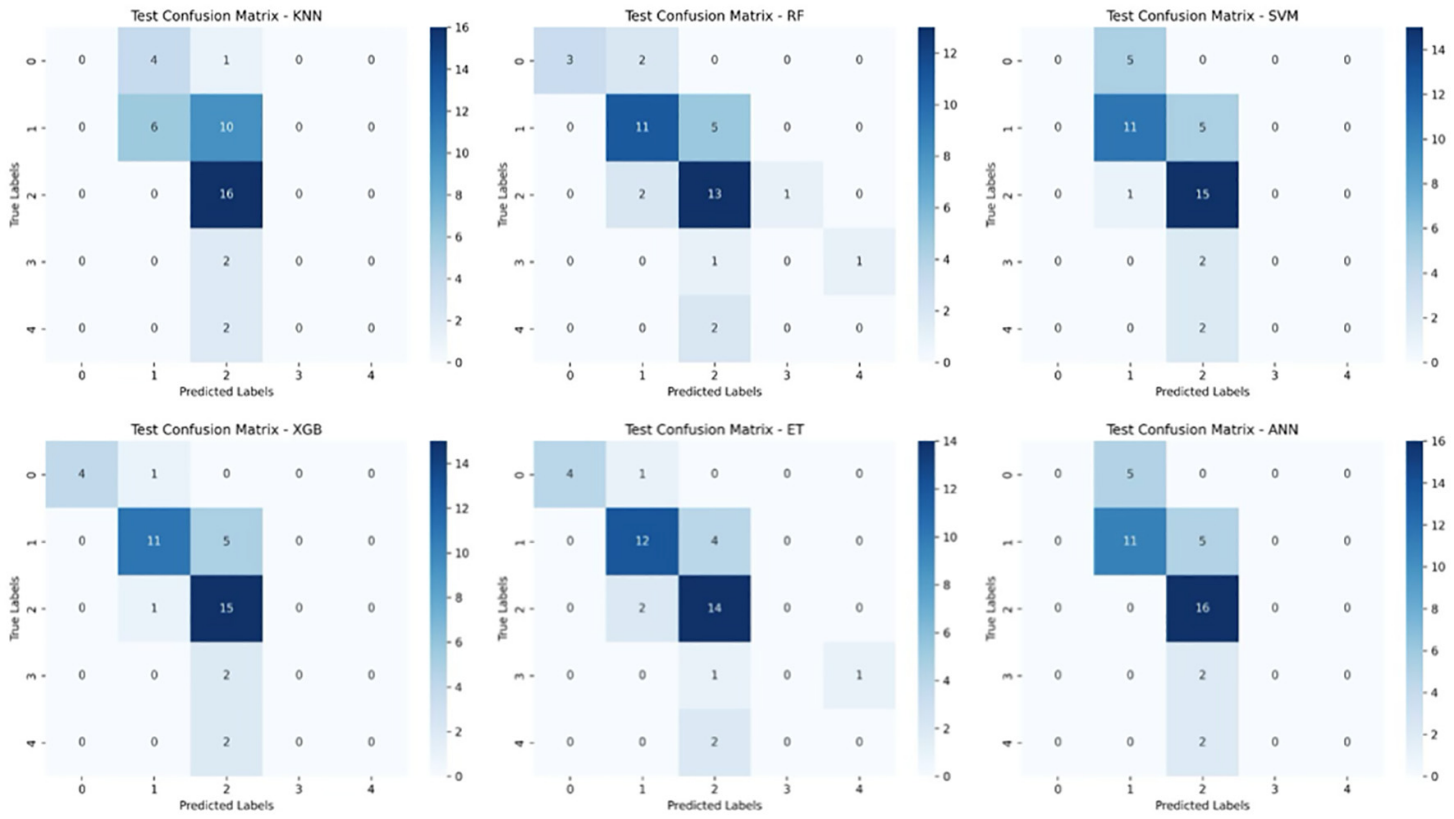
Test Set Confusion Matrices for Six Machine Learning Models. This figure displays the confusion matrices for six machine learning models (KNN, Random Forest (RF), Support Vector Machine (SVM), XGBoost, Extra Trees (ET), and Artificial Neural Networks (ANN)) when tested on unseen data for glioblastoma survival prediction. The matrices compare the true survival classes (y-axis) against the predicted labels (x-axis) for five survival categories.

To further assess model performance, we analyzed the confusion matrices for both the training and test sets (**Figures 6**, **7**). These matrices provide a detailed breakdown of how well each classifier distinguishes between the five survival classes (0–2 months, 3–8 months, 9–18 months, 19–24 months, and >24 months).

**Figure 8:**
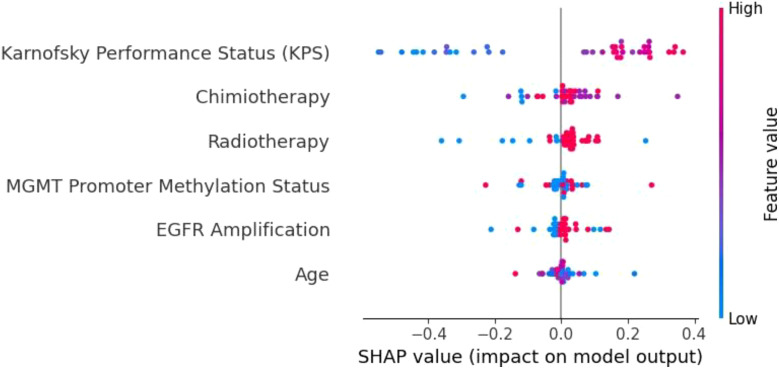
SHAP summary plot for the KNN model. The limited differentiation of SHAP values across features highlights KNN’s weak ability to prioritize key variables, contributing to its poor generalization and frequent misclassifications.

**Figure 9:**
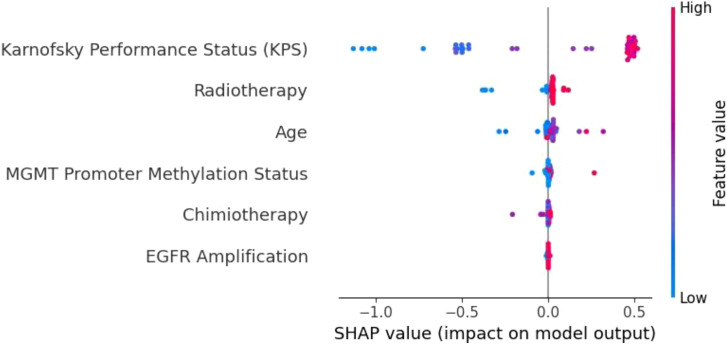
SHAP summary plot for the XGBoost model. KPS is the most influential feature, followed by radiotherapy, age, and MGMT promoter methylation. The distinct separation of SHAP values demonstrates XGBoost’s capacity to effectively prioritize important prognostic factors.

**Figure 10:**
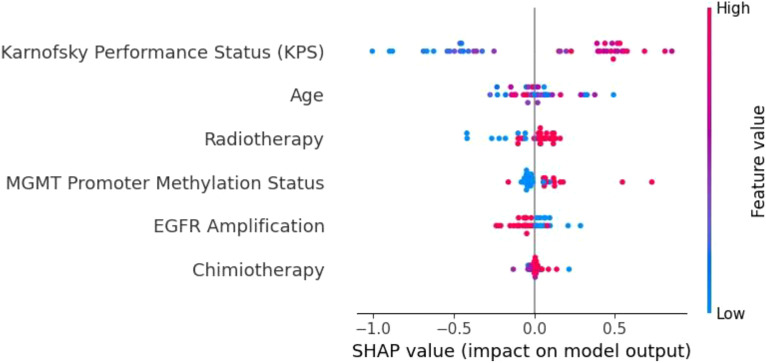
SHAP summary plot for the Extra Trees model. KPS is the dominant feature, followed by age, radiotherapy, and MGMT promoter methylation. The Extra Trees model demonstrates strong feature differentiation, contributing to its robust predictive performance in glioblastoma survival prediction.

To understand the decision-making processes and feature prioritization of the evaluated models, we applied SHAP analysis to the KNN, XGBoost, and Extra Trees models. The SHAP summary plots (**Figures 8**–**10**) provide a comprehensive evaluation of the models’ interpretability, highlighting their respective strengths and limitations.

The KNN model (**Figure 8**) demonstrated limited feature differentiation, reflecting its inherent weakness in handling high-dimensional and imbalanced data. While KPS emerged as the most influential feature, the lack of distinct separation among other variables indicates that KNN struggled to assign proper weight to important factors such as MGMT promoter methylation and radiotherapy. This deficiency is consistent with KNN’s frequent misclassifications and its poor generalization to the test set. Given its reliance on local data density and sensitivity to sparse distributions, KNN is not well-suited for complex clinical datasets like those involving glioblastoma patients. As such, we recommend that simpler models like KNN be replaced by ensemble-based methods for tasks involving high-dimensional and heterogeneous medical data.

In contrast, the XGBoost model (**Figure 9**) exhibited strong and consistent feature prioritization, which explains its superior predictive performance. KPS was identified as the most critical factor, followed closely by radiotherapy, age, and MGMT promoter methylation. These results align with established clinical knowledge, as higher KPS scores and positive MGMT promoter methylation are associated with improved survival outcomes in glioblastoma patients.

The clear separation of SHAP values highlights XGBoost’s capacity to integrate diverse clinical and molecular data, effectively capturing non-linear interactions between features. This ability to discern complex patterns and prioritize clinically meaningful variables is a key factor behind its high accuracy and generalization capability.

The Extra Trees model (**Figure 10**) performed comparably to XGBoost, further validating the strength of ensemble-based models in this context. KPS once again emerged as the dominant feature, underscoring its central role in survival prediction. The model effectively leveraged other important variables, including age, radiotherapy, and MGMT promoter methylation, demonstrating its ability to capture both treatment-related and biological factors. The differentiation among SHAP values shows that the Extra Trees model can robustly identify key contributors to patient outcomes, even in the presence of class imbalance and data heterogeneity. This adaptability makes it a reliable tool for clinical decision support, particularly when high interpretability and predictive performance are required.

Overall, the SHAP analysis underscores the superiority of ensemble-based methods like XGBoost and Extra Trees over simpler models such as KNN. By effectively prioritizing clinically significant features and accommodating complex interactions, these models offer both high accuracy and interpretability—critical components for integrating machine learning into personalized glioblastoma treatment strategies. Future enhancements, such as incorporating longitudinal patient data or multi-omics integration, could further improve their predictive capabilities, ensuring even greater clinical utility.

## 6 Discussions

This study delves into the application of advanced ML techniques to predict OS in GBM patients, offering a comprehensive integration of clinical, molecular, and treatment-related data. Our findings illuminate the strengths of ML models in capturing the complexity of GBM prognosis while identifying key challenges and opportunities for refinement.

### 6.1 Model performance and insights

XGBoost emerged as the most robust model in our analysis, achieving a mean ROC-AUC of 0.90 and an accuracy of 0.73. Its ability to manage heterogeneous and non-linear data interactions aligns with its established success in oncology applications. Gradient boosting techniques, like XGBoost, have gained recognition for their versatility in handling high-dimensional datasets. For instance, studies integrating clinical, transcriptomic, and radiomic data have demonstrated XGBoost’s capacity to identify nuanced survival patterns, underscoring its adaptability to complex datasets.

Other models, such as SVM and ensemble approaches like ET and RF, also demonstrated strong predictive power, with SVM achieving a mean ROC-AUC of 0.84 and ET at 0.82. SVM’s ability to perform well on smaller datasets is particularly relevant given the limited cohort sizes often encountered in GBM research. Ensemble methods excel in feature prioritization, providing interpretable insights into key prognostic variables such as MGMT promoter methylation and KPS. These findings echo recent studies that highlight ensemble models as vital tools for identifying clinically actionable predictors in oncology.

Conversely, ANN and KNN showed limited predictive capacity. While ANNs have shown promise in larger datasets due to their ability to recognize intricate patterns, their performance can falter with smaller, imbalanced datasets like ours. KNN’s relatively poor performance, with an accuracy of 0.54, suggests it may not be suitable for high-dimensional datasets with sparse or unevenly distributed features. These results align with existing literature emphasizing the limitations of these approaches in specific contexts.

### 6.2 Innovations and methodological contributions

A strength of this study lies in the rigorous preprocessing methods employed, including label encoding and MinMax scaling, which ensured uniformity across variables. By categorizing OS into distinct survival classes, the study enabled a more granular stratification of patients. This approach mirrors recent advancements in predictive oncology, where discrete outcome modeling enhances the precision of survival estimates.

The fine-tuning of hyperparameters across models further underscores the methodological rigor. For instance, optimizing parameters such as learning rates, tree depth, and kernel selection significantly improved model accuracy. These strategies are increasingly regarded as essential in developing reliable predictive frameworks, as evidenced in contemporary GBM prognosis research.

### 6.3 Challenges and limitations

Despite its strengths, the study faced challenges that are emblematic of GBM research. The dataset’s class imbalance, particularly among long-term survival categories, limited the models’ ability to accurately predict outcomes for these underrepresented groups. Addressing this imbalance requires innovative solutions, such as synthetic data generation using techniques like GANs or oversampling methods like SMOTE. Recent studies utilizing synthetic data have shown promise in enriching underrepresented classes while preserving the underlying data distribution (19–21).

Another hurdle is the interpretability of complex ML models. While algorithms like XGBoost deliver high accuracy, their “black-box” nature limits transparency, which can impede clinical adoption. Emerging tools such as SHAP and LIME offer potential solutions by elucidating feature contributions, enabling clinicians to trust and act upon model predictions. Incorporating these interpretability frameworks into future iterations of our models would bridge the gap between accuracy and usability.

Furthermore, the single-cohort nature of the dataset necessitates external validation to ensure generalizability. Multi-institutional collaborations and federated learning approaches, which allow for model training across decentralized datasets while preserving patient privacy, represent a promising avenue for addressing this limitation. Such methodologies have shown great potential in recent multi-center oncology studies.

### 6.4 Clinical implications and future directions

The findings of this study highlight the transformative potential of ML in GBM prognosis. Accurate survival predictions have profound implications for patient care, from guiding individualized treatment strategies to identifying candidates for experimental therapies and optimizing resource allocation. Models like XGBoost not only deliver precise predictions but also underscore the prognostic value of variables such as MGMT promoter methylation, age, and KPS, reinforcing their relevance in clinical decision-making.

Future research should explore integrating longitudinal data to enable dynamic survival predictions that evolve alongside patient trajectories. Incorporating multi-omics data, such as proteomic and epigenomic profiles, into ML pipelines could further refine prognostic accuracy. Hybrid models that balance the interpretability of simpler algorithms with the predictive power of advanced techniques like gradient boosting could offer the best of both worlds, ensuring both accuracy and clinical usability.

## Data Availability

The raw data supporting the conclusions of this article will be made available by the authors, without undue reservation.
